# Risk factors for incident prostate cancer in a cohort of world trade center responders

**DOI:** 10.1186/s12888-019-2383-1

**Published:** 2019-12-10

**Authors:** Sean A. P. Clouston, Peifen Kuan, Roman Kotov, Soumyadeep Mukherjee, Patricia Thompson-Carino, Evelyn J. Bromet, Benjamin J. Luft

**Affiliations:** 1 Department of Family, Population, and Preventive Medicine, Program in Public Health, Stony Brook Medicine, Health Sciences Center, #3-071, Nichols Rd., Stony Brook, NY 11794-8338 USA; 2 Department of Applied Mathematics, Stony Brook University, Stony Brook, NY USA; 3 Department of Psychiatry, Stony Brook Medicine, Stony Brook, NY USA; 4 Program in Public Health, Stony Brook Medicine, Stony Brook, NY USA; 5 Department of Pathology, Stony Brook Medicine, Stony Brook, NY USA; 6 World Trade Center Health and Wellness Program, Department of Medicine, Stony Brook Medicine, Stony Brook, NY USA

**Keywords:** World trade center, Posttraumatic stress disorder, Prostate Cancer, Cancer epidemiology

## Abstract

**Background:**

Despite a relatively young average age and no routine screening, prostate cancer is one of the most common cancers in men who worked at the World Trade Center (WTC) following the 9/11/2001 disaster. This study evaluated whether re-experiencing stressful memories of a traumatic event was associated with prostate cancer incidence.

**Methods:**

Participants were males from one clinical center that monitors the health of first-responders (*N* = 6857). Monitoring began in July 2002 and occurs annually but does not include prostate cancer screening. Severity of physical exposures and of re-experiencing memories and stress responses were measured at study enrollment using standardized and validated methods in all participants. The outcome was incidence of diagnosed prostate cancer after enrollment (*n* = 68). Bivariate analyses provided age-adjusted incidence rates (aIR). Cox proportional hazards modeling was used to calculate incidence; hazards ratios (HR) were reported.

**Results:**

The mean age of responders on 9/11/2001 was 37.9 years. Prostate cancer incidence was lowest in responders with no re-experiencing stress (aIR = 250.83/100,000 person-years, [233.41–268.25]) and highest in responders with severe re-experiencing stress (aIR = 818.49/100,000 person-years, [801.07–835.91]). Cox proportional hazards regression revealed that re-experiencing the stressful events of 9/11/2001 was associated with increased prostate cancer incidence (HR = 1.96 [1.26–3.05], *P* = 0.003), even upon adjusting for confounders.

**Conclusions:**

This is the first study to identify a positive association between re-experiencing a traumatic event and prostate cancer incidence. Our results are consistent with recent rodent model evidence demonstrating a direct biological link between stress pathways and prostate tumorigenesis and offer new hypotheses in the causality of prostate cancer.

## Background

It has previously been reported that men who responded to the 9/11/2001 events and worked on-site at the World Trade Center (WTC) (hereafter: “responders”) are experiencing a higher than expected incidence of prostate cancer [1–4] and of more aggressive disease as later stage diagnoses when compared to the NY state cancer registry [3]. To date, however, efforts to identify possible carcinogens and physical exposures have found no link between the WTC physical exposures and the observed increase in incidence of prostate cancer [4]. During these events, however, responders were exposed to a host of emotional stressors both during the event and in the months that followed and many responders, in the years since, have reported having persistent and chronic post-traumatic stress disorder (PTSD) [5].

While a few groups have provided evidence supporting a link between stress and prostate cancer, recent work from Zahalka et al. [6] provides important experimental evidence for a direct link between the nervous system and cancer in the prostate gland. Using genetic models of prostate cancer and knock out studies, Zahlaka et al. [6] demonstrated that autonomic nerves within the prostate microenvironment were required for the activation of a metabolic switch necessary for exponential tumor growth. This and previous mouse model studies of prostate cancer implicate a direct role for intra-prostatic sympathetic nerves and adrenergic signaling in angiogenesis and tumor growth. Moreover, the *β2*-adrenergic receptor (ADRB2) is implicated in differentiation of prostate tumor cells to neuroendocrine-like cells, an established histologic feature of aggressive prostate cancer. As reviewed by Braadland et al. [7], an emerging potential consequence of sympathetic signaling to prostate cancer, is evidence for prostatic nerve derived adrenergic stimuli on adrenergic receptors in the tumor microenvironment and direct effects on tumorigenesis. ADRB2 is the most abundant receptor for adrenergic signals in prostate luminal cells. Collectively, these data provide biological plausibility that chronic hyperactivation of the sympathetic nervous system while being chronically exposed to a severe stressor, and its deregulation, may have direct effects at the prostate tissue level.

Traumatic exposures, as occurred with the World Trade Center attack, can cause a similar process where individuals re-experience stress-inducing memories of their most severe stressors for a long time after a traumatic event [8]. Importantly, when an individual is exposed to traumatic events, the brain activates a host of neural systems including the noradrenergic nucleus located in the locus coeruleus in the midbrain [9]. Critically, the locus coeruleus helps to regulate both emotional responses to external *stimuli* [10], and activation of physical locomotion as a response to stress [10]. The adrenergic response also plays a key role in the activation of the amygdaloid complex, resulting in increased recognition and improved storage of emotionally-intense experiences [11]. For example, activation of α1-adrenergic receptors engages the hippocampus to increase capacity for long-term storage of traumatic memories for later retrieval and processing, while activation of the ADRB2 facilitates later retrieval of stressful memories [12].

During re-experienced memories, an individual can react as though he or she is experiencing the stress. This includes activation of the hypothalamic-pituitary-adrenal axis [13] and resultant release of norepinephrine, which is critical to the fight or flight response and activation of ADRB2. In addition to effects on the amygdala and hippocampus to amplify and process stressful memories [12], glucocorticoids are activated to signal that the stress response is no longer necessary [14] thereby activating glucocorticoid receptors [15]. Importantly, prostate cancer pathogenesis has been mechanistically linked to β-adrenergic and glucocorticoid receptors, with evidence that dysfunctions in these pathways may be associated with more aggressive forms of the disease [16].

Based on this evidence, we hypothesized that increased re-experiencing stress severity may explain the increased prostate cancer incidence observed in WTC responders.

## Methods

### Study population

This study utilized population-level data to examine risk of prostate cancer in a clinical center that monitors World Trade Center (WTC) responders residing in Long Island, NY [17]. In this population of WTC responders, more than 90% were working at the time of 9/11/2001, and the majority were employed by the New *York Police Department*. All monitored male WTC responders who consented to participate in research studies were eligible for this study (*N* = 6918). To reduce the risk of reverse causation, 37 cases diagnosed since 9/11/2001 but prior to the first monitoring visit were excluded from analyses. Responders with missing data on confounders were excluded from analyses (*n =* 62); those who were excluded had symptoms of PTSD similar to (*p* = 0.987) those who were included in the study. The final analytic sample therefore included 6857 eligible male responders contributing a total of 47,261.2 person-years of information.

### External reference population

The included WTC responders resided on Long Island, NY. For comparison purposes to the local population risk and cancer stage, we have utilized age-adjusted prostate cancer incidence rates derived from the NY state cancer registry for the most recent years available (2010–2014) [18]. To account for geographic variability in incidence of prostate cancer, sensitivity analyses also calculated an inverse case-weighted average for Nassau and Suffolk counties.

### Assessment of prostate cancer incidence and severity

Prostate cancer (ICD-10: C61.9) was diagnosed during regular clinical visits and confirmed by clinicians at the Centers for Disease Control and Prevention. Date of diagnosis and Gleason score were recorded. Histology and cancer staging were recorded using a standard coding scheme [19]. For these analyses, cancer stage was categorized into early (Stage I/II) and late-stage (Stage III/IV). In some cases, a prostate cancer diagnosis followed another cancer diagnosis; in these cases, the date of the prostate-specific cancer diagnosis was recorded alongside the type of other cancer.

### Assessment of PTSD symptoms

PTSD symptoms and symptom severity was measured at study enrollment using the exposure-specific version of the PTSD checklist that indexed symptoms specifically to the WTC events (PCL) [20]. Individuals rated the extent to which they were bothered by seventeen PTSD symptoms in the past month. Ratings were made on a scale ranging from 1 (not at all) to 5 (extremely). Since animal studies implicated re-traumatization accompanied by a heightened physiologic stress response, we here focused on the five core re-experiencing symptoms (the first five symptoms in the PCL) including: being bothered by disturbing memories, dreams, reliving the stressful experience, upsetting reminders of the event, and having a physical reaction to the reminders. The five re-experiencing symptoms were averaged to provide a symptom severity score using a validated methodology [21] and then, to facilitate comparison of effect sizes with other risk factors, the index was rescaled to range from zero (0; no symptoms) to one (1; severe symptoms). Because there is no consistent guidance on inclusion of continuous variables in regression analyses, for effect size comparisons we also report hazards ratios (HR) for PTSD reflecting a one-standard deviation (HR_SD_) and interquartile range (HR_IQR_) increase in PTSD symptoms.

### Assessment of covariates

Age in years on 9/11/2001 was used. Body mass was calculated using observed height in centimeters and weight in kilograms. Race/ethnicity was categorized as non-Hispanic White, non-Hispanic Black, non-Hispanic other, and Hispanic. *WTC exposure severity* was assessed using a structured history and answers formed the basis for an index created using a validated measure relying upon weights determined using a Delphi technique to combine pulmonary exposure measures experienced at the WTC both on 9/11/2001 and in the months after as described [22].

### Screening information

Since the risk of prostate cancer diagnosis is influenced by the utilization of prostate-specific antigen (PSA)-based prostate cancer screening, there is a potential for bias if PTSD symptom severity influences the risk of PSA-testing rates. Cancer screening for responders in monitoring clinics follows the recommendations made by the *American Cancer Society* [23]. Since *American Cancer Society* guidelines advise against routine screening for prostate cancer, it is not provided to responders at monitoring clinics. As such, the incidence of PSA testing is unobserved. To capture the potential for increased screening among those with PTSD symptoms, this study examined whether PTSD symptom severity was associated with increased risk of referral for any other cancer screening. For men, cancer screening is routinely provided for colorectal and lung cancers. To assess the potential for screening bias we therefore examined whether PTSD symptom severity was associated with increased risk of receiving any cancer screening referral and, secondarily, whether PTSD symptoms were associated with increased adherence to cancer referrals. Cause-specific cancer referrals and referral completion were available between 01/01/2016–12/31/2017.

### Statistical analysis

Means and standard deviations, as well as percentages were used for descriptive purposes. Crude and age-specific incidence rates were calculated. In descriptive analyses, an exponential trend curve was overlaid on age-specific rates using least squares regression. Age-adjusted incidence rates were calculated using the direct standardization approach weighted to the 2000 U.S. standard population. Age-adjusted incidence rates were used to calculate standardized incidence ratios (SIR), defined as the ratio of observed incidence rates and expected incidence based on location-specific cancer registry data. To examine differences in prostate cancer staging, relative risks (RR) were calculated comparing to NY state registry data. In all cases, 95% confidence intervals were reported.

Log-binomial regression was used to model risk for receiving a referral for cancer screening when adjusting for multiple confounders because cancer screening is a common outcome [24]. Age and sex-adjusted risk ratios (RR), 95% confidence intervals were estimated, and *p*-values were provided.

The risk of receiving up to three cancer-screening options, conditional on being referred for cancer screening was modeled using negative binomial regression. Negative binomial regression was used in lieu of Poisson regression because rates were over-dispersed (α = 0.53), a condition whereby the variance is larger than the mean [25].

The risk of incident cancer was modeled using Cox proportional hazards regression [26]. Cox models are a semi-parametric form of survival modeling that reliably models survival without incident prostate cancer under the conditions that competing illnesses do not result in decreased risk of incidence and in the event that the proportional hazards assumption is met. Responders were deemed to have entered the study on 9/11/2001. Time until prostate cancer diagnosis was the outcome. Responders were censored at their diagnosis or at their last visit date. Hazards ratios and 95% confidence intervals, as well as the cumulative hazards curve, were reported. Schoenfeld residuals were used to test the proportional hazards assumption.

## Results

The monitoring program population was in early midlife (average age = 37.9 ± 1.66 years [range = 16–75]) in 2001 and the majority were non-Hispanic White (Table 1). Those who had diagnoses of prostate cancer were older, had more severe PTSD symptoms, and differed in Race/Ethnicity than were those without prostate cancer.

**Table 1:** Characteristics of the Stony Brook University World Trade Center Health and Wellness Program population

	No Prostate cancer		Prostate Cancer Diagnosis		
Patient Characteristic	Mean	SD	Mean	SD	P-value
Age at 9/11/2001	37.8	8.2	49.8	8.6	< 0.001
Re-experiencing symptoms	0.3	0.4	0.5	0.5	0.003
WTC Exposure Severity	14.5	4.5	14.5	4.4	0.919
Weeks worked at the WTC	5.3	4.5	5.4	4.6	0.814
Body Mass	30.9	5.2	29.8	4.2	0.066
	%		%		P-value
Race/Ethnicity					0.033
White	72.2%		81.0%		
Black	3.3%		5.1%		
Asian/Pacific Islander	0.8%		–		
American Indian/Alaskan Native	0.2%		–		
Multi-Race	0.4%		1.3%		
Other/Unknown Race	16.0%		2.5%		
Hispanic	7.1%		10.1%		

**Note:** WTC: World Trade Center; SD: Standard Deviation; P-values were derived from Student’s t-test for continuous variables, and from Chi-Squared tests for categorical variables

A total of 68 eligible responders were diagnosed with prostate cancer after their first monitoring visit (Crude Incidence Rate (IR) = 152.34/100,000 95% C.I. = [120.92–191.93]). The age-adjusted incidence rate was (aIR = 267.36, [249.94–284.78]). Compared to SEER data, WTC responders were at increased risk of developing prostate cancer (SIR = 1.95, [1.55–2.44]). Sensitivity analyses relying only on Nassau and Suffolk counties, where monitored responders reside, revealed similar overall results (SIR = 1.66, [1.31–2.07]).

In addition to prostate cancer, seven responders had at least one additional comorbid cancer totaling 11 cancers, including cancers of the lung/bronchus (*n* = 2), urinary bladder (n = 2), colorectal (n = 2), kidney and renal pelvis (n = 2), non-Hodgkin’s lymphoma (*n* = 1), stomach (n = 1), and thyroid (n = 1). Of these, most responders had only one additional cancer, while one responder had two and one had three.

Of those with diagnoses of prostate cancers, 67 (98.5%) had adenocarcinoma while one had an acinar adenocarcinoma. Prostate cancers were staged as I (*n =* 6), II (*n =* 25), III (*n =* 35), and IV (*n =* 1), with one unknown, revealing that 31/68 (45.6%) were identified in the early stage. Compared to the NY state average (Stage I/II = 79.4% in 2014), WTC responders’ prostate cancers were more likely to be identified at Stage III/IV (RR = 2.49, [1.98–3.13]).

**Fig. 1:**
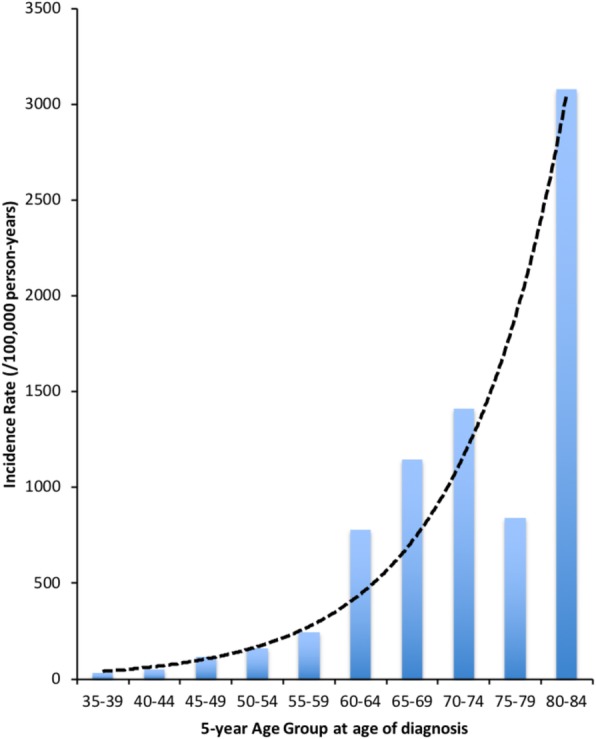
Age-specific incidence rates. Blue bars provide age-specific incidence rates. Black dotted line shows smoothed risk curve

Incidence rates of prostate cancer increased sharply with age (Fig. 1). Incidence rates also differed by re-experiencing symptom severity. Those with low re-experiencing symptom severity scores ranging from (0.00–0.10; *n* = 3100; 24 cancers) had age-adjusted incidence rates of 250.83 [233.41–268.25], which increased to 291.55 [2.74.13–308.97] in medium (score = 0.20–0.49; *n* = 1947; 26 cancers), and 818.49 [801.07–835.91] in high re-experiencing symptom severity (0.50–1.00; *n* = 1810; 29 cancers) categories respectively.

Results from cancer screening analyses revealed that half (51.5%) of all screening referrals in 2016/17 were adhered with. Multivariable analyses revealed that re-experiencing symptom severity was not associated with the risk of receiving a cancer screening referral (aRR = 0.97 [0.73–1.29], *P* = 0.833), and that re-experiencing symptom severity was not associated with increased risk of adherence risk when patients were received a screening referral (aRR = 1.06 [0.80–1.41], *P* = 0.672).

**Fig. 2:**
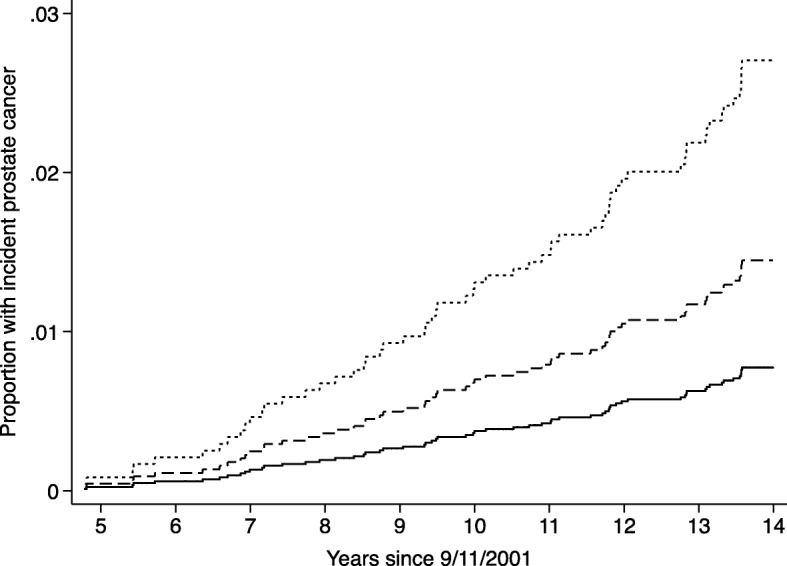
Cumulative hazards curve examining prostate cancer incidence by PTSD symptom severity. **Note:** Re-experiencing symptom severity is shown as no symptom severity (solid line; re-experiencing symptom scores = 0.00–0.10) compared to moderate scores (dashed line; scores ranging from 0.11–0.49) and responders with severe re-experiencing symptom severity (dashed line; scores ranging from 0.50–1.00)

Bivariable and multivariable incidence analyses revealed that re-experiencing symptom severity was associated with increased incidence of prostate cancer (HR = 3.87 [1.60–9.36], *P* = 0.021) and the accompanying cumulative hazards curve was provided (Fig. 2). This level of risk is equivalent to an HR_IQR_ = 1.40 [1.12–1.75] or a standardized effect size of HR_SD_ = 1.32 [1.10–1.57]. Bivariate results were corroborated in multivariable analyses revealed similar results (Table 2). This level of risk is equivalent to an aHR_IQR_ = 1.40 [1.12–1.75] or a standardized effect size of aHR_SD_ = 1.32 [1.10–1.57]. These analyses also identified age with increased risk of prostate cancer, and reporting “Other” race as being associated with reduced risk of prostate cancer. Of potential interest, risk of incident prostate cancer appeared more strongly associated with PSD symptoms in men from other Races (HR = 10.56 [1.54–72.33]), though the interaction effect was not significant in these data (HR = 4.15, *P* = 0.256). Schoenfeld residuals tests revealed that the study did not violate proportional-hazards assumptions.

**Table 2:** Multivariable adjusted hazards ratios and 95% Confidence Intervals derived from Cox proportional hazards regression examining predictors of prostate cancer incidence since 9/11/2001

Characteristic	aHR	95% C.I.	*P*
Re-experiencing symptom severity	3.50	[1.37–8.96]	0.009
Age on 9/11/2001	1.14	[1.12–1.17]	0.000
Exposure severity	0.99	[0.86–1.14]	0.871
Body Mass	0.98	[0.94–1.02]	0.344
Race/Ethnicity			
White	1.00		
Black	1.35	[0.49–3.72]	0.563
Other	0.20	[0.06–0.63]	0.006
Hispanic	1.59	[0.76–3.32]	0.218

**Note**: aHR: multivariable-adjusted hazard ratio

## Discussion

In this study, data from an academic monitoring program for WTC responders revealed a positive association between re-experiencing symptom severity following a traumatic event and risk of prostate cancer. Replicating earlier efforts in a prior study showing that included multiple monitoring clinics, we found that the study sample is at heightened risk (SIR = 1.95 [1.55–2.44]) for prostate cancer compared to state cancer registries. Further, we provide the first evidence of a small positive association between increased levels of re-experiencing stress and incidence of prostate cancer (HR = 1.96 [1.26–3.05], *P* = 0.003). To our knowledge, this is the first prospective study to link any psychiatric condition with incidence of cause-specific cancer.

Heightened incidence of prostate cancer has been previously reported in veterans [27] and early in WTC responders [1, 2]. For the latter, similar results have been reported across the multiple cohorts of WTC exposed populations and differences in screening do not appear to account for the consistently elevated risk in these groups [3, 4]. Despite extensive efforts, no plausible environmental exposure has been identified to account for the higher rates in veterans or WTC responders. For example, efforts to link Agent Orange to the increased risk of prostate cancer are equivocal [27], as have been efforts to link WTC exposure severity with elevated incidence [28]. Other very large studies of prostate cancer risk factors have also failed to identify any consistent risk factors for prostate cancer and the etiology of prostate cancer has remained elusive.

More recently, animal model studies support direct effects of the nervous system in prostate cancer biology and raise the possibility of stress effects on nervous system derived signals in prostate cancer. Studies in humans that support this causal hypothesis are limited. Findings from epidemiological studies on the role of stress in prostate cancer, are contradictory and limited by lack of sufficiently large studies with quality measures on stress exposures and too few outcomes to stratify on cancer type [29–32]. Only a single prospective study reported a positive association between perceived level of stress and risk of prostate cancer over a five-year period [33] while other studies that have linked screening-induced anxiety to higher PSA levels are themselves prone to symptom bias [34, 35]. In cancer patients, psychological distress is associated with worse outcomes [29, 31, 36, 37], which may suggest an underlying biological effect of stress in tumor behavior though psychosocial impacts on access and use of cancer treatment remain potential confounders. Interestingly and consistent with the animal data is limited epidemiologic evidence that β-blockers used to inhibit the action of ADRB2 are associated with lower prostate cancer mortality [38]. Perhaps the strongest epidemiologic evidence for ‘stress’ and prostate cancer are findings from the Physicians’ Health/Health Professionals Follow-Up Study [39] that compared gene expression profiles in 254 prostate cancers and 120 normal tissues from cohort participants. In their study, Lu *et.al.* demonstrated that stress-related signaling pathways, including adrenergic and glucocorticoid genes and their associated pathways, were positively associated with a more lethal form of prostate cancer and worse survival.

This work builds on evidence that re-experiencing symptoms provoke the body’s fight or flight response mechanisms linking PTSD symptoms to deregulated glucocorticoid and β-adrenergic receptor activation in the periphery. Important to our study, most responders with re-experiencing symptoms (used here) do not attain the level of severity and of functional limitations necessary for a clinical diagnosis of PTSD. For example, only 10% of responders are currently diagnosed with PTSD [5], whereas 32.9% are categorized as having medium levels of re-experiencing symptoms and 21.7% have high PTSD symptomatology in this study. Future efforts to replicate the observed increased prostate cancer risks associated with PTSD in our study should consider the role of PTSD symptom types in populations and prostate cancer risk.

### Strengths and limitations

This is one of the only studies to examine the potential for posttraumatic re-experiencing symptom severity to increase the risk of prostate cancer. Unlike many studies, this study is notable because re-experiencing symptoms were measured at enrollment and therefore, by design, prior to the diagnosis of incident prostate cancer. Diagnoses were identified following strict guidelines, and adherence to these guidelines was independently verified by staff at the Centers for Disease Control and Prevention.

A primary limitation is the relative youth of the study cohort as a whole (aged 54 on average in January 2018) since 57% of all prostate cancers are diagnosed in men aged 65 and older [40]. Additionally, the cohort is primarily non-Hispanic White with relatively respectable jobs who served in a response effort to a unique but severely stressful event and live in Long Island, NY. These factors limit the generalizability of this result in meaningful ways. Notably, there is good evidence to suggest that African Americans are at increased risk of prostate cancer, which may make that population uniquely vulnerable to additional risk due to stress-based exposures, and hypothesis that was not interrogated here due to the small number of cancers overall. In our data, African Americans were not at significantly increased risk although there was a non-significant trend towards a stronger association between re-experiencing symptoms and incidence of prostate cancer in African Americans. Improving generalizability and extending results to minorities will require larger, more diverse samples and/or longer follow-up periods. There is limited information about the role of screening in this population. While being a clinical cohort, it is worthwhile noting that the monitoring program has never provided screening as a matter of course to WTC responders. While it is plausible that screening may be more common off-site among responders as a group, screening and severity indicators interrogated herein suggest that this is unlikely. Specifically, when compared to state registry data suggesting that 20.6% of prostate cancers are diagnosed at Stages III/IV, we found that 40/78 (51.3%) of prostate cancers were identified in Stages III/IV (RR = 2.49 [1.98–3.13]). Additionally, we found no suggestion that PTSD symptom severity might increase risk of cancer screening referral and completion, and also found that completion was relatively low. Finally, there are a number of other conditions including lifetime risk of mood disorders [41], major depressive disorder [42], and manic disorders [43] that have been linked with increased risk of PTSD after a trauma and may, therefore, play a heretofore unknown role in determining the risk of prostate cancer. Together, these results suggest that while there remains the potential for screening bias, the extent of this bias may be limited.

## Conclusions

This study identified a novel association between increased PTSD re-experiencing symptom severity and increased incidence of prostate cancer in a cohort of WTC responders who were prospectively monitored for cancer as part of an academic monitoring program. The biological plausibility of our observation is supported by findings from experimental models of prostate tumorigenesis and if replicated, may represent an important step towards understanding pathogenesis of prostate cancer in traumatized populations.

## Data Availability

Outcome data used in this paper include exact ages, date of interview, specific diagnostic codes, and dates of diagnosis that are identifiable and cannot therefore be uploaded to publicly-accessible websites. Access to these data can be provided upon receipt of a written data request. Data used in this paper can also be independently accessed by approved researchers through the WTC Data Coordination Center located at the Icahn School of Medicine at Mount Sinai (icahn.mssm.edu/about/departments/environmental-public-health/research/wtc-data-center).

## Abbreviations

95% C.I.: 95% confidence interval
ADRB2: *β2*-adrenergic receptor
aIR: age-adjusted incidence rate
HR: Hazards Ratio
ICD: international statistical classification of diseases and related health problems
IQR: inter-quartile range
IR: Crude Incidence Rate
NY: New York
PCL: Posttraumatic stress disorder checklist
PSA: prostate-specific antigen
PTSD: posttraumatic stress disorder
RR: relative risk
SD: Standard deviation
SIR: Standardized incidence ratio
WTC: World Trade Center
